# Stool and Ruminal Microbiome Components Associated With Methane Emission and Feed Efficiency in Nelore Beef Cattle

**DOI:** 10.3389/fgene.2022.812828

**Published:** 2022-05-17

**Authors:** Bruno G. N. Andrade, Flavia A. Bressani, Rafael R. C. Cuadrat, Tainã F. Cardoso, Jessica M. Malheiros, Priscila S. N. de Oliveira, Juliana Petrini, Gerson B. Mourão, Luiz L. Coutinho, James M. Reecy, James E. Koltes, Adhemar Z. Neto, Sérgio R. de Medeiros, Alexandre Berndt, Julio C. P. Palhares, Haithem Afli, Luciana C. A. Regitano

**Affiliations:** ^1^ Embrapa Southeast Livestock, São Carlos, Brazil; ^2^ Department of Computer Science, Munster Technological University, MTU/ADAPT, Cork, Ireland; ^3^ Department of Molecular Epidemiology, German Institute of Human Nutrition Potsdam-Rehbrücke (DIfE), Nuthetal, Germany; ^4^ Federal University of São Carlos, UFSCar, Brazil; ^5^ Department of Animal Science, University of São Paulo/ESALQ, Piracicaba, Brazil; ^6^ Department of Animal Science, Iowa State University, Ames, IA, United States; ^7^ Embrapa Agriculture Informatics, Campinas, Brazil

**Keywords:** association, archaea, bacteria, biomarkers, *Bos indicus*, feed efficiency, methane emission

## Abstract

**Background:** The impact of extreme changes in weather patterns on the economy and human welfare is one of the biggest challenges our civilization faces. From anthropogenic contributions to climate change, reducing the impact of farming activities is a priority since it is responsible for up to 18% of global greenhouse gas emissions. To this end, we tested whether ruminal and stool microbiome components could be used as biomarkers for methane emission and feed efficiency in bovine by studying 52 Brazilian Nelore bulls belonging to two feed intervention treatment groups, that is, conventional and by-product-based diets.

**Results:** We identified a total of 5,693 amplicon sequence variants (ASVs) in the Nelore bulls’ microbiomes. A Differential abundance analysis with the ANCOM approach identified 30 bacterial and 15 archaeal ASVs as differentially abundant (DA) among treatment groups. An association analysis using Maaslin2 software and a linear mixed model indicated that bacterial ASVs are linked to the host’s residual methane emission (RCH_4_) and residual feed intake (RFI) phenotype variation, suggesting their potential as targets for interventions or biomarkers.

**Conclusion:** The feed composition induced significant differences in both abundance and richness of ruminal and stool microbial populations in ruminants of the Nelore breed. The industrial by-product-based dietary treatment applied to our experimental groups influenced the microbiome diversity of bacteria and archaea but not of protozoa. ASVs were associated with RCH_4_ emission and RFI in ruminal and stool microbiomes. While ruminal ASVs were expected to influence CH_4_ emission and RFI, the relationship of stool taxa, such as *Alistipes* and Rikenellaceae (gut group RC9), with these traits was not reported before and might be associated with host health due to their link to anti-inflammatory compounds. Overall, the ASVs associated here have the potential to be used as biomarkers for these complex phenotypes.

## Background

The Anthropocene Epoch is marked by the continuous degradation of the biosphere promoted by human activities, culminating in the ongoing climate and environmental crisis (Williams et al., 2015; Cook et al., 2016). One of the current challenges is to mitigate its effects and sustain an ever-growing human population by developing methods for efficient food production. Cattle farming, a valuable source of animal protein, is alone responsible for a significant environmental impact due to the occupation and degradation of land for pasture, contamination of water sources by cattle manure (Sahoo et al., 2016), and the emission of methane (CH_4_) produced by enteric fermentation, a greenhouse gas 28 times stronger than CO_2_ (Pachauri and Mayer, 2015).

Key phenotypes for the reduction of the meat industry’s environmental and economic burden, such as feed efficiency and methane emission, are linked to interactions between the host and its associated microbial communities, also known as the microbiota (Guan et al., 2008; Chang et al., 2019). Although studies targeting these phenotypes have been published over the years (Mudadu et al., 2016; Pszczola et al., 2018; Lassen and Difford, 2020), only recently the microbiota started to be considered as an important subject to increase efficiency and reduce costs and the environmental impact of cattle farming (Shi et al., 2014; Noel et al., 2019).

These microorganisms can shape their host biology through beneficial interactions and influence health, development, and immune system modulation (Flint et al., 2007; Belkaid and Hand, 2014). However, most of these microorganisms are elusive and utterly unknown to science due to inherent difficulties in cultivation procedures (Solden et al., 2016). Nowadays, it is possible to access microorganisms’ genomic material directly and investigate their identity, distribution, relatedness, and functionality using approaches from the meta-omics field, such as deep sequencing metagenomics, metabarcoding, and metatranscriptomics (Gilbert and Dupont, 2011).

The microbiome structure of the Nelore Brazilian beef cattle has gained the attention of the scientific community in the past years, being the subject of different studies (de Oliveira et al., 2013; Lopes et al., 2021), including a previous study by our research group (Andrade et al., 2020) in which we investigated the microbiome profiles of two segments of 26 Nelore bulls’ gastrointestinal tract (GIT). We showed that a significant part of the stool archaeal population co-occurred with the rumen archaeal population, suggesting the use of stool as a proxy for the rumen archaeal population.

Herein, we extended that the study by the introduction of an additional experimental group under a different diet and compared the microbiome populations from two distant sections of the Nelore GIT-rumen and rectal ampulla to 1) identify the impact of the dietary treatment on the microbiome diversity and abundance; 2) identify associations between microbiome components and phenotypes, such as residual CH_4_ emission (RCH_4_) and residual feed intake (RFI).

## Methods

### Experimental Design

All experimental procedures were conducted following animal welfare guidelines and were approved by the EMBRAPA Livestock Science Ethics Committee on Animal Experimentation, São Carlos, São Paulo (Protocol No. 09/2016). The experimental population consisted of animals born in 2014, and the experiment was conducted at the feedlot facility of “Embrapa Pecuária Sudeste”. It lasted 105 days, which included 15 days for animal adaptation to the feedlot, 30 days for growth, and 60 days for animal finishing. Animals were divided into two groups based on dietary treatment. The first experimental group (conventional group, n = 26) consisted of animals fed with a conventional diet based on corn silage, corn, soybean meals, rumen-protected fat, and urea as a concentrate, as described in Andrade et al. (2020). The second experimental group (by-product group, n = 26) replaced concentrates with the industrial by-products citrus pulp, corn germ, corn germ oil meal, and peanut shell meal. In both treatment groups, animals received mineral supplements, active dry yeast, virginiamycin, and monensin.

Feedlots were divided based on the dietary treatment and initial weights, with heavyweight and lightweight animals grouped separately. The facility has collective stalls with an automatic feeding system (GrowSafe Systems Ltd., Airdrie, Alberta, Canada), in order to collect data regarding live weight and daily food consumption. The animals were then sent for slaughter at 23–24 months of age, following the Guidelines for Humane Handling, Transport, and Slaughter of Livestock. All animal data used in this study are available in Supplementary Table S1.

### Host DNA Extraction and Genotyping

5 mL of blood samples were collected from each animal, and DNA extractions were performed by a salting-out method (Meo et al., 2009). DNA concentration was measured by spectrophotometry, and quality was verified by the 260/280 optical density ratio, followed by integrity inspection through agarose gel electrophoresis. All animals were genotyped using the GGP *Bos indicus* 50 k. Genotypes were called in the Illumina GenomeStudio software. We applied the following filtering parameters with PLINK software (Chang et al., 2015): 1) SNP call rate ≤95%, 2) SNP minor allele frequency (MAF) ≤ 5%, 3) animals with ≥10% missing genotypes, and 4) SNPs that did not pass the HWE test (*p* ≤ 0.001). A total of 41,869 SNPs of all 52 animals were available for further analysis.

### Phenotypes

#### Residual Feed Intake

Individual dry matter intake (DMI, kg/d) was obtained by the difference between the weight of the diet provided and refusal, and average daily gain (ADG, kg/d) was estimated by linear regression of body weight (BW) on days in feedlot. Residual feed intake (RFI, kg/d) was computed as the residuals from the regression of DMI on mid-test BW_0.75_ and ADG (Koch et al., 1963). The metabolic body weight (MBW, kg) was obtained with the following equation: MBW = BW^0.75^. The contemporary group (CG) was defined as the weighing group and the slaughter group, which were considered fixed effects by MIXED procedure of the SAS statistical program (SAS Institute, Cary, NC, United States, 2011), according to the following equation:

$$\text{D}\text{M}{\text{I}}_{\text{i}}={\text{β}}_{0}+{\text{β}}_{1}\left(\text{A}\text{D}{\text{G}}_{\text{i}}\right)+{\text{β}}_{2}\left(\text{M}\text{B}{\text{W}}_{\text{i}}\right)+{\text{β}}_{3}\left(\text{C}{\text{G}}_{\text{i}}\right)+\text{R}\text{F}{\text{I}}_{\text{i}},$$ (1)

where DMI_*i*_ is the dry matter intake predicted for animal *i*; ADG_*i*_ is the average daily gain of animal *i*; MBW_*i*_ is the metabolic body weight of animal *i*; β_*0*_ is the regression intercept; β_1_ is the partial regression coefficient of ADG; β_2_ is the partial regression coefficient of MBW; and β_3_ is the partial regression coefficient of CG, and RFI_*i*_ is the RFI of animal *i* (Koch et al., 1963).

### Residual Methane Emission

The methane emission was measured during the finishing period in the feedlot using the GreenFeed system (Clock Inc., Rapid City, SD, United States). The residual methane emission (RCH_4_) was obtained by the regression of methane emission using individual DMI (Donoghue et al., 2016), and CG as covariables in the MIXED procedure of SAS statistical program (SAS Institute, Cary, NC, United States, 2011), according to the following equation:

$$\text{M}{\text{E}}_{\text{i}}={\text{β}}_{0}+\text{β}1\left(\text{D}\text{M}{\text{I}}_{\text{i}}\right)+{\text{β}}_{2}\left(\text{C}{\text{G}}_{\text{i}}\right)+\text{r}\text{e}\text{sidual}\;\text{error},$$ (2)

where ME_*i*_ is the methane emission predicted for animal *i*; DMI_*i*_ is the dry matter intake predicted for animal *i*; β_0_ is the regression intercept; β_1_ is the partial regression coefficient of DMI; β_2_ is the partial regression coefficient of CG; and, as proposed by Donoghue et al. (2016), the model residual error of animal *i* was considered as the residual methane emission (RCH_4_).

### Microbiota Sample Collection and Processing

After the finishing phase, approximately 10 g of stool was obtained from each animal 2 weeks before slaughtering, and 50 ml of rumen content was collected immediately after slaughter. All samples were frozen in liquid nitrogen and permanently stored at −80°C before analysis. DNA extraction was performed using the Quick-DNA™ Fecal/Soil Microbe Miniprep Kit (ZYMO Research Corp., Irvine, CA), using 150 mg of each sample and following the standard protocol. PCR target amplification for the bacterial and archaeal 16S rRNA and protozoal 18S rRNA coding genes was performed using the primers 341-b-S-17F and 341-b-S-17F (Klindworth et al., 2013), Ar915aF and Ar1386R (Kittelmann et al., 2013), and Reg1320R and RP841F (Henderson et al., 2015), respectively, following Andrade et al. (2020). Amplicons were sequenced in an Illumina Miseq platform (2 × 250 bp) using the Illumina V3 sequencing kit at the ESALQ Genomics Center (Piracicaba, SP, Brazil).

### Data Retrieval, Pre-Processing, and Analysis

In addition to the dataset generated in this study, raw reads generated by our previous study with bulls fed conventional diet were retrieved from the SRA database [accession number PRJNA525838] and processed to infer the impact of dietary treatments and to search for association with phenotypes.

Raw reads from conventional and by-product groups were filtered for quality (>Q25) and trimmed at positions 220 (forward) and 175 (reverse) using QIIME 2 version 2018.8 (Bolyen et al., 2019). We selected these positions based on aggregation plots provided by QIIME 2. The filtered data was submitted to the DADA2 package to generate amplicon sequence variants (ASVs) with the option just-concatenate and exclude chimeric sequences (Callahan et al., 2016). Bacterial sequences were annotated using the SILVA database version 132 (Quast et al., 2013), archaeal sequences using the Rumen and Intestinal Methanogen database (RIM-DB) (Seedorf et al., 2014), and protozoa using a curated database containing protozoa 18S rRNA gene sequences (Kittelmann et al., 2015). We used the resulting ASV table to determine alpha (number of ASVs and the Shannon–Wiener index) and beta diversities (unweighted UniFrac distance) with QIIME 2.

### Statistical Analysis

We contrasted the microbiome of groups submitted to different dietary treatments using the Analysis of Composition of Microbiomes (ANCOM) version 2.1 (Kaul et al., 2017), with significance values adjusted for multiple tests using the Benjamin–Hochberg method (α < 0.05). We applied a conservative W-statistic (W-statistic cutoff = 0.9) in which an ASV was considered as differentially abundant if its composition varied when compared to 90% of the rest of the dataset, being the W-value the number of times the model rejected the null hypothesis for a given ASV across two groups. ANCOM is a statistical approach that compares Aitchison’s centered log-ratio transformed abundances of each ASV individually with all the remaining ASVs without any distributional assumptions (Kaul et al., 2017).

ASV abundances were tested for associations with animal phenotypes, such as RCH_4_ and RFI phenotypes using the Microbiome Multivariable Association with Linear Models (Maaslin) version 2 (Mallick et al., 2021). Maaslin is a multivariate model developed for the microbiome data analysis, as it considers the compositional nature of these datasets. The analysis was adjusted for treatment and contemporary groups using the following formula:

$$\text{m}0:\;\text{C}\text{L}\text{R}\left(\text{A}\text{S}\text{V}\right)∼\text{P}\text{h}\text{e}\text{n}\text{o}\text{t}\text{y}\text{p}\text{e}\left(\text{R}\text{F}\text{I}\;\text{o}\text{r}\;\text{R}\text{C}{\text{H}}_{4}\right)+\text{D}\text{i}\text{e}\text{t}+\text{C}\text{G}+\text{r}\text{esidual}\;\text{error},$$ (3)

where CLR is the ASV abundance transformed using the centered log-ratio method, and e is the residual error. ASVs identified as significant by the Maaslin analysis, and that was prevalent in more than 20% of animals with a minimum abundance of 0.001%, had their effect on the phenotypic variation (response variable) tested by using the linear mixed model implemented in the lme4qtl package (Ziyatdinov et al., 2018). Genetic relatedness was included as a random effect in the form of a kinship matrix to rule out the population structure effect in the phenotypic variation, jointly with diet and CG fitted as fixed effects. We built the genomic relatedness matrix using the AGHmatrix package (Amadeu et al., 2016) of R (v. 4.0.3), based on the genomic matrix proposed elsewhere (VanRaden, 2008).

A likelihood ratio test was used to contrast models (m1 and m2) to investigate the ASV impact on the phenotype. Our model can be described by the following formula:

m1: phenotype (RFI or RCH_4_) ∼ Diet + CG + Kinship Matrix + residual error.

m2: phenotype (RFI or RCH_4_) ∼ centered log-ratio (ASV) + Diet + CG + Kinship Matrix + residual error.

Significant values for all models were adjusted for multiple tests using the false discovery rate (FDR) method (*α*<= 0.05).

## Results

### Microbiome Composition

The sequencing of microbiome rDNA amplicons from ruminal and stool samples of the by-product group yielded a total of 10,573,763 paired-end reads (4,628,604 paired-end reads for bacteria, 4,443,390 for archaea, and 1,501,769 for protozoa), reaching 20,241,296 paired-end reads with the addition of sequencing data from animals fed conventional diet. After quality control and singleton exclusion, we identified a total of 4,519 bacterial ASVs (2,680 ruminal ASVs and 1,839 stool ASVs), 1,023 archaeal ASVs (421 ruminal ASVs and 602 stool ASVs), and 151 ruminal protozoa ASVs across treatments. Rarefaction curves based on the alpha diversity metrics of the Shannon–Wiener index (diversity) reached a plateau, which indicated that additional sequences would not likely result in additional features (Supplementary Figure S1).

**FIGURE 1:**
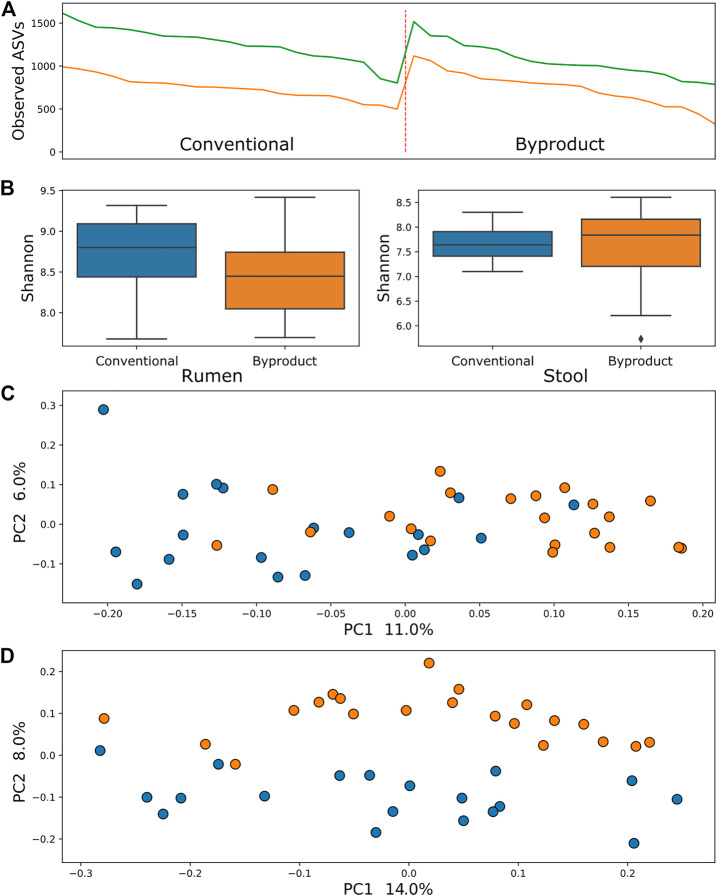
**(A)** Comparison between observed ASV (amplicon sequence variants) metric between treatment groups showed no significant difference in the bacterial population of animals submitted to different diets in both rumen (green line) and stool (yellow line). **(B)** Shannon index comparisons showing a significant difference (*p* < 0.01) in the richness of bacteria from the rumen microbiome. **(C)** PCoA using the rumen microbiome unweighted UniFrac distance showing a tendency of clustering of samples from conventional group (blue) and B (orange). **(D)** PCoA using the stool microbiome unweighted UniFrac distance showing an almost linear separation of samples from animals fed conventional diet (blue) and by-product diet (orange).

**FIGURE 2:**
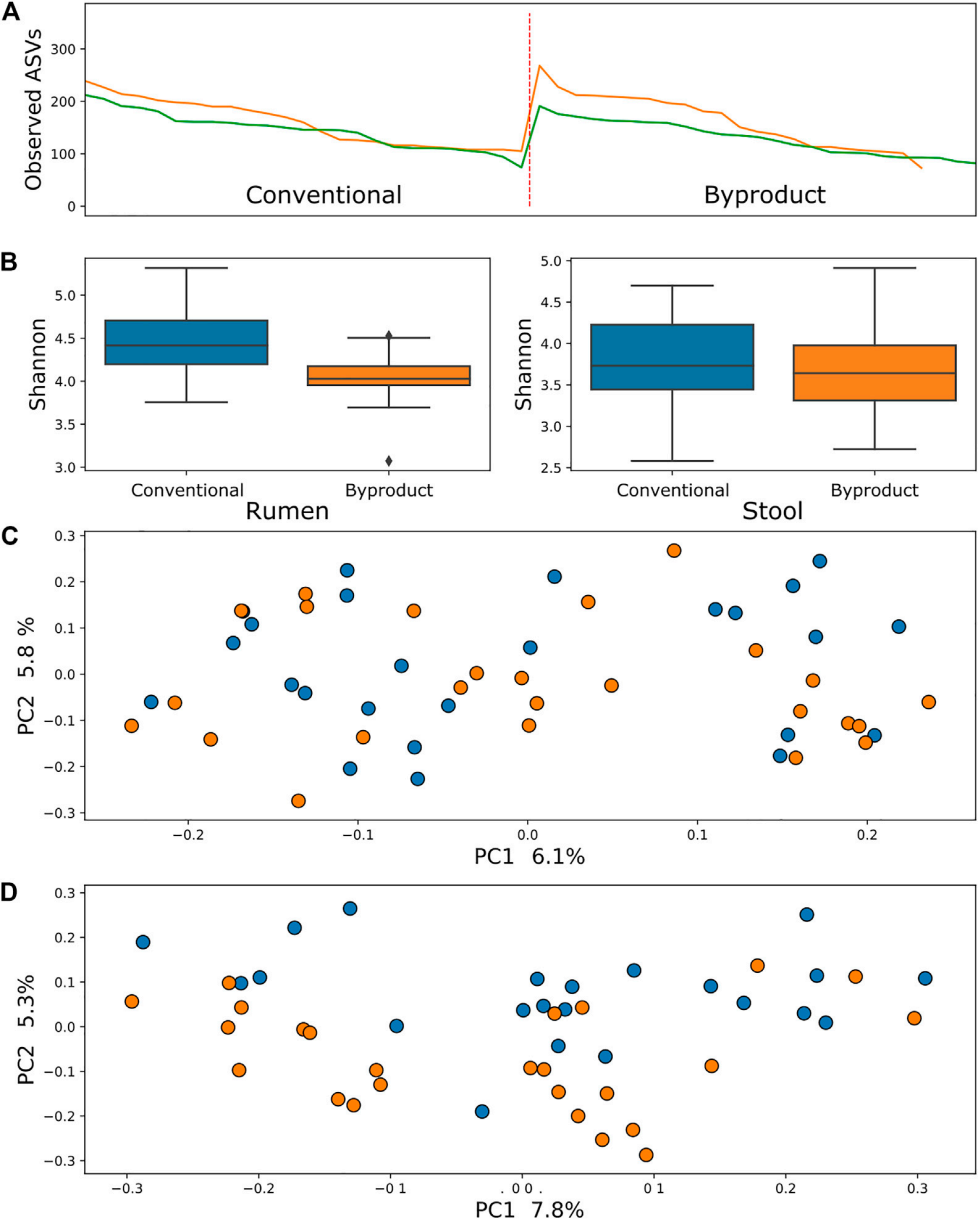
**(A)** Comparison between observed ASV (amplicon sequence variants) metric between treatment groups showed no significant difference in archaea populations of animals submitted to different diets in both rumen (green line) and stool (yellow line). **(B)** Shannon index comparisons showing a significant difference (*p* < 0.01) in the richness of archaea in the rumen microbiome. **(C)** PCoA using the rumen microbiome unweighted UniFrac distance showing no significant difference between groups (*p* > 0.1). **(D)** PCoA using the stool microbiome unweighted UniFrac distance showing a tendency of clustering of samples from animals fed conventional diet (blue) and by-product diet (orange).

Comparison of samples from different treatment groups using alpha diversity metrics (observed ASVs and Shannon–Wiener indexes) by the Kruskal–Wallis testing method revealed that rumen bacterial microbiome was significantly more abundant and richer in animals fed the conventional diet than those fed the by-product diet (*p* = 0.006 and *p* = 0.04, respectively), whereas the ruminal archaea diversity was richer (*p* = 0.0004) but not more abundant. There was no significant difference between diets when contrasting alpha diversity metrics of stool samples. Conversely, comparisons of the beta diversity metric unweighted UniFrac using the PERMANOVA approach revealed that samples of archaea and bacteria tended to form two significant clusters, which represented the treatment groups (adjusted *p* < 0.01) (Figures 1, 2), a tendency most pronounced in stool populations. Protozoa populations showed no significant differences between treatment groups (Supplementary Figure S2).

### Differentially Abundant ASVs in Dietary Treatment Groups

We applied ANCOM to investigate the influence of dietary treatments in the microbiome composition at the ASV level. Seventeen ruminal ASVs of bacterial origin were differentially abundant (DA) with higher abundance in the conventional group, from which the most prominent were classified as Bacteroidales (group F082) (ASV 20 and 23), Christensenellaceae (ASV 112), Pedosphaeraceae families (ASV 145), and the genus *Succiniclasticum* (ASV 170). Ten DA ASVs had higher abundance in the by-product group, of which the most abundant were classified as *Succiniclasticum* (ASV 97), *Acetitomaculum* (ASV 116), Lachnospiraceae family (ASV 247), *Fibrobacter* (ASV 96), and *Succinivibrio* genus (ASV 118) (Supplementary Figure S3). Also, three stool ASVs were DA in our experimental groups; one was classified as a member of the family Rikenellaceae (ASV 361) and was more abundant animals fed conventional diet, while the ASV 332, classified as a member of the family Prevotellaceae, and the ASV 526, classified as the genus *Oscillibacter*, were more abundant in animals fed the by-product diet (Supplementary Figure S4).

Eight archaeal ASVs were DA among treatment groups in the rumen microbiome. Four ASVs classified as *M. gottschalkii* (ASVs 1, 2, 13, and 11), one as *M. ruminantium* (ASV 23), and one ASV belonging to the family Methanomassiliiicoccaceae (ASV 36) were all more abundant in animals fed conventional diet. In contrast, one classified as *M. ruminantium* (ASV 4) and the other as Methanosphaera (group ISO3-F5) (ASV 33) were more abundant in the by-product group (Supplementary Figure S5). Seven archaeal ASVs were DA in the stool microbiome. From these, the ASVs classified as *M. gottschalkii* (ASVs 2, 13, and 11) and *M. smithii* (ASV 28) were more abundant in animals fed a conventional diet. At the same time, *M. ruminantium* (ASV 4) and Methanosphaera (group ISO3-F5) (ASVs 5 and 33) were more abundant in animals fed by-product diet (Supplementary Figure S6). No ASVs of protozoa origin were observed as DA for any biome or group.

### Association Between Bacterial ASVs Identified in the Nelore GIT and RCH_4_

**FIGURE 3:**
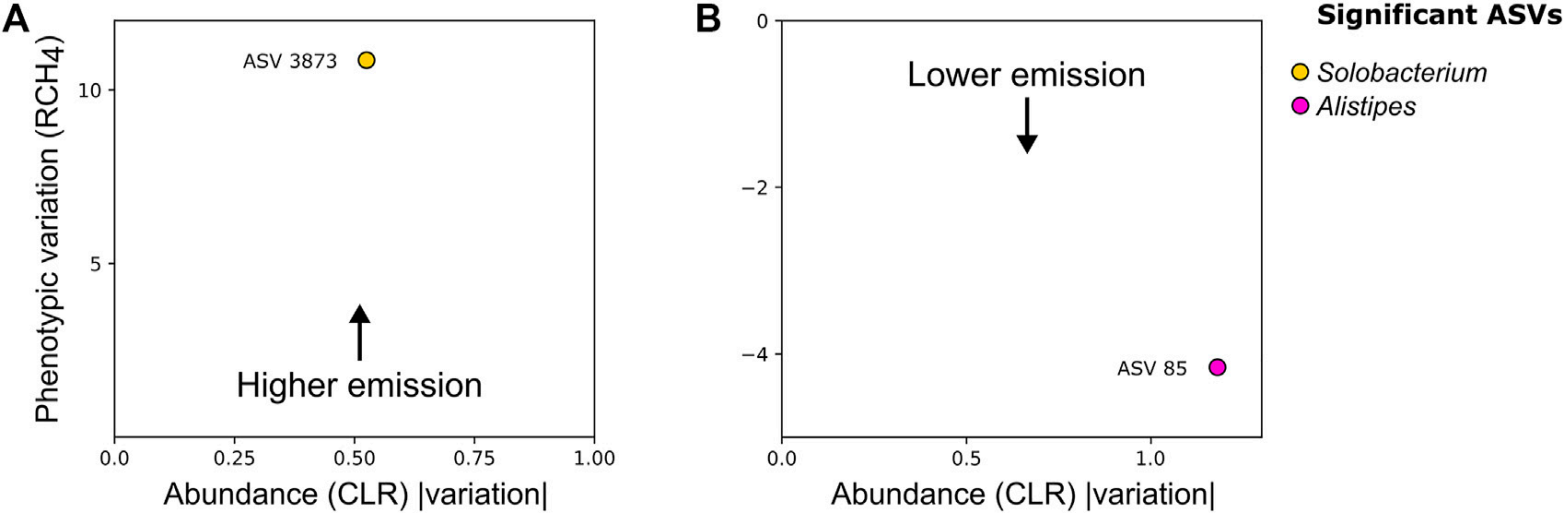
Standardized beta coefficient for the RCH_4_ trait *versus* the module abundance (CLR) variation of amplicon sequence variants (ASV) in **(A)** rumen; **(B)** stool. Both the phenotypic variation and ASV abundance variation were retrieved from the beta coefficients of mixed models and Maaslin2 GLM regressions. Taxonomic information generated by QIIME 2 was included.

We applied a generalized linear model (GLM) within the Maaslin2 framework to investigate ASVs associated with RCH_4_ emission variation. This analysis allowed us to identify significant associations between bacteria and RCH_4_ in both environments. A second model was used to validate the direction and strength of association of ASVs identified by Maaslin2. We identified a single bacterial ASV associated with High-RCH_4_ (High emission) in the rumen, the ASV 3873 (coef_CLR = 0.54, coef_RCH_4_ = 0.48), classified as a *Solobacterium* (Figure 3A), and one associated with Low-RCH_4_ (Low emission) in the stool environment, the ASV 85 (coef_CLR = −1.18, coef_RCH_4_ = −0.48), classified as *Alistipes* (Figure 3B). There were no significant associations between RCH_4_ emission and archaea or protozoa ASVs.

### Association Between Bacterial ASVs in the Nelore GIT and Feed Efficiency

**FIGURE 4:**
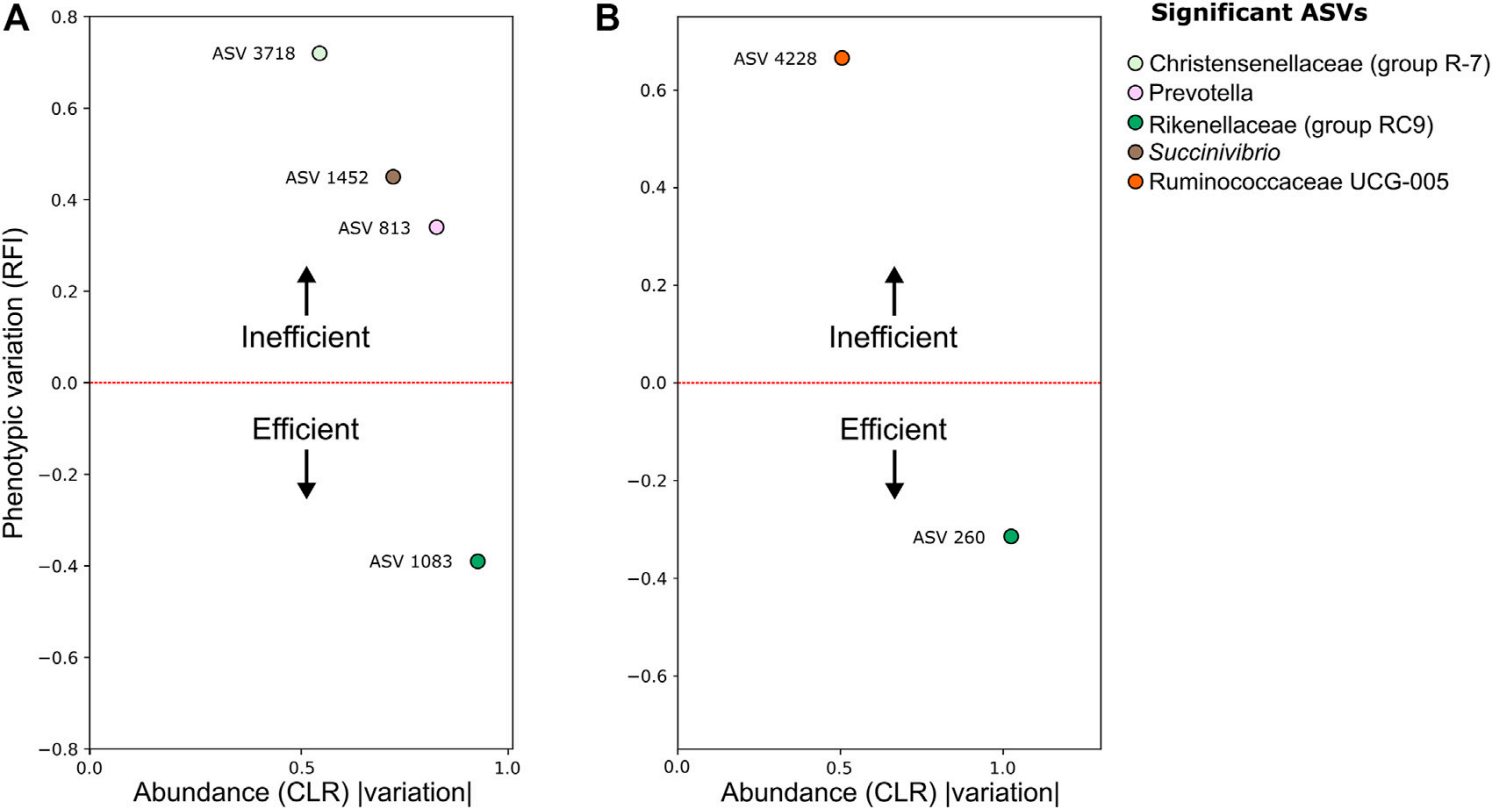
Standardized beta coefficient for the RFI trait *vs.* the module abundance (CLR) variation of amplicon sequence Vvriants (ASV) in **(A)** rumen; **(B)** stool. Both the phenotypic and ASV abundance variations were retrieved from the beta coefficients of mixed models and Maaslin2 GLM regressions. Taxonomic information generated by QIIME 2 was included in the legend.

Association analysis between RFI and microbiome components was performed using the same GLM and mixed models described for the RCH_4_ analysis. We identified four bacterial ASVs associated with RFI in the rumen environment, three of these associated with feed inefficient (High-RFI) and one ASV associated with feed efficient animals (Low-RFI) (Figure 4). We also identified one ASV associated with High-RFI and one with Low-RFI in the stool environment (Figure 2).

Among those associated with High-RFI, were the ASV 3718 (coef_CLR = 0.54, coef_RFI = 0.54), classified as Christensenellaceae (gut group R-7), the ASV 1452 (coef_CLR = 0.71, coef_RFI = 0.49), classified as *Succinivibrio*, ASV 813 (coef_CLR = 0.82, coef_RFI = 0.45), classified as *Prevotella* in the rumen, and the ASV 4228 (coef_CLR = 0.49, coef_RFI = 0.50), classified as Ruminococcaceae UCG-005, in stool environments. The ASV 1083 (coef_CLR = −0.91, coef_RFI = −0.48) and ASV 260 (coef_CLR = −1.00, coef_RFI = −0.46), both classified as Rikenellaceae RC9, were significant in Low-RFI (Figure 4) in rumen and stool environments, respectively. There were no significant associations between RFI, and archaea or protozoa ASVs.

## Discussion

Our previous study extensively explored the microbiome structure from two different sections of the Nelore cattle GIT under a single dietary treatment (Andrade et al., 2020). Herein, we expand this study by introducing a new experimental group under a different diet and related the microbiome to production phenotypes. We compared these groups to investigate the impact of the diet on microbial abundance and diversity, and the contribution of individual ASVs on complex phenotypes, such as RFI and RCH_4_.

### The Microbiome Structure Is Affected by Feed Composition

Analysis of alpha diversity metrics showed that both bacteria and archaea only differed in the rumen environment, being less rich in animals of the by-product group. This richness difference could be explained by the presence of citrus pectin on the by-product diet formulation. Citrus pectin is a polysaccharide that can selectively stimulate the microbiome, affecting its composition (Larsen et al., 2019), and is linked to the decrease of *Prevotella copri*, a bacteria associated with rheumatoid arthritis in humans (Pianta et al., 2017). Also, PCoA analysis of the unweighted UniFrac beta diversity metric highlighted distinct clusters in treatment groups for bacteria (rumen and stool microbiomes) and archaea (stool microbiome), but not for protozoa. Altogether, these results indicate the diet as an essential microbiome modulator for bacteria and archaea populations, in agreement with previous studies in which different diets and feed components’ impact on microbiome diversity was evaluated (Dill-McFarland et al., 2019; Popova et al., 2019). Such differences in the microbiome composition had little to no impact on the phenotypes studied herein, in agreement with the lack of diet effect on phenotypes. We hypothesize, however, that this lack of observable effect could be explained by the microbiome functional redundancy, in which the taxonomic composition can vary between individuals, but its functional capacity is conserved (Tian et al., 2020). However, this hypothesis resides outside the scope of this paper, and further studies have to be implemented to test it.

Differential abundance analysis with individual bacterial ASVs revealed a significant impact of the dietary treatment in the bacterial populations of both environments. In the rumen, the ASVs classified as belonging to the Christensenellaceae family and the *Prevotella* and *Fibrobacter* genera were identified as more abundant in animals fed a conventional diet. These microorganisms are known producers of short-chain fatty acids (SCFAs), such as acetate, butyrate, and propionate (Neumann et al., 2017; Waters and Ley, 2019). Besides, ASVs classified within genera known to produce succinate and propionate, that is, *Succiniclasticum* and *Succinivibrio* (Hespell, 1992; van Gylswyk, 1995), were identified as more abundant in animals fed by-product diet. Differently from acetate and butyrate production, which increases H_2_ concentration in the rumen (Wolin, 1960), propionate is an electron acceptor end product of rumen fermentation and a viable alternative to methanogenesis to decrease the ruminal H_2_, a process known as H_2_ sink (Ungerfeld, 2015; Wang et al., 2018). This alternative H_2_ sink process decreases energy loss caused by methanogenesis and the resulting surplus of feed energy has the potential of diluting the host maintenance costs, increasing its feed efficiency.

The three DA ASVs identified in the stool samples were classified as bacteria that commonly inhabit the hindgut, including the *Oscillibacter* genus and Prevotellaceae family, both more abundant in animals fed by-product diet, and Rikenellaceae family, more abundant in animals fed conventional diet (Marounek and Duskova, 1999; Chen et al., 2011; Lee et al., 2012). The identification of a small number of DA ASVs in the stool microbiome is consistent with the previous alpha diversity analysis, in which there was no significant difference in both abundance and richness among experimental groups.

The dietary treatment also significantly impacted the archaea populations, *e.g.*, an increased abundance of ASVs classified as *M. gottschalkii* in both rumen and stool environment of animals fed conventional diet, and *M. ruminantium* in both environments of animals fed by-product diet. A study on sheep with contrasting phenotypes for CH_4_ emission found a higher abundance of the archaea *M. gottschalkii* in the higher emitter group and *M. ruminantium* in the lower emitter group (Shi et al., 2014).

### Phenotypic Associations Indicate Biomarkers for RCH_4_ Emission in the GIT Microbiota

The GLM model analysis identified two bacterial ASVs associated with the RCH_4_ phenotype in the rumen and stool microbiomes. This reduced number of significant results was expected since this phenotype is complex and caused by different microbe–microbe and host–microbiome interactions. In addition, the microbiome functional redundancy may also play an essential role in it, reducing the likelihood of pinpointing a biomarker using metabarcoding alone.

Regarding the rumen microbiome, an ASV classified as belonging to the *Solobacterium* genus was identified as the most abundant bacterial ASV in animals with High-RCH_4_. This genus was first described in 1999 (Kageyama and Benno, 2000) and had the species *Solobacterium moorei* as its sole representative. This bacterium is an important producer of volatile sulfur compounds (VSC) such as hydrogen sulfide (H_2_S) in the human oral microbiome (Stephen et al., 2014). This compound was previously identified *in vitro* as having inhibitory properties against methane oxidation (Lee et al., 2015), preventing methanotrophs from metabolizing CH_4_ by using it as a carbon source, which could have, in turn, contributed to the increase in CH_4_ concentration.

In the stool microbiome, the ASV belonging to the *Alistipes* genus was associated with the decrease of the RCH_4_. This genus comprises Gram-negative and anaerobic bacteria commonly identified in the bovine GIT microbiome (Dowd et al., 2008; Holman and Gzyl, 2019). Like other members of the Rikenellaceae family, bacteria from this genus produce acetate and propionate, both fatty acids with anti-inflammatory properties in the gut of humans and chickens (Polansky et al., 2015; Parker et al., 2020).

Although we were not able to identify a direct link between anti-inflammatory compounds and methane production, researchers have been describing the potential use of different species of seaweed as a feed component to reduce the enteric methane production in dairy and beef cattle (Machado et al., 2014; Abbott et al., 2020). These seaweed species, especially the red seaweed, are rich to some extent in alkaloids, a nitrogenous compound with microbiome modulator capabilities and anti-inflammatory activity (Abbott et al., 2020). Although this link is hypothetical, the relationship between these taxa with methane emission and their biomarker/probiotic potential has to be further investigated using more layers of metagenomic information, such as metagenomes and metatranscriptomes. Another possible mechanism for this association comes from the observation of an increase in peristaltic movements in humans with higher abundance of the genus Alistipes (Scarpato et al., 2019; Sugitani et al., 2021). If this effect holds for ruminants, it could affect rumen emptying, thus providing a mechanism for the reduction in methane emission.

Surprisingly, we could not identify archaeal ASVs associated with RCH_4,_ in both environments. Known significant contributors to methane production, species of archaea belonging to the *Methanobrevibacter* genus, such as *M. gottschalkii* and *M. ruminantium*, were highly abundant in the rumen microbiome of both experimental groups, with the diet having a significant impact on the archaea population structure. Still, archaeal ASVs failed to reach the significance threshold regarding this phenotype.

### Feed Efficiency Is Linked to SCFA Producers in Both Environments

The ASVs identified as associated with High-RFI (decreasing the feed efficiency) in the rumen environment were taxonomically classified as *Prevotella* and *Succinivibrio* genus, and Christensenellaceae (gut group R-7). These results agreed with a recent study with rumen samples from Nelore cattle, in which OTUs classified as belonging to these same genera were linked to High-RFI (Lopes et al., 2021). Species from the genus *Prevotella* have a major role in the digestion of complex polysaccharides, such as cellulose and hemicellulose, and have been identified as abundant in both spectra of feed efficiency in cattle (Carberry et al., 2012; Hernandez-Sanabria et al., 2012; Myer et al., 2015), suggesting that the effect of *Prevotella* in the feed efficiency phenotype in cattle is species-specific (Perea et al., 2017).

*Succinivibrio dextrinosolvens*, the only representative species of the *Succinivibrio* genus, presented an increased relative abundance in the rumen fluid of High-RFI Hereford × Aberdeen Angus steers (Hernandez-Sanabria et al., 2012) and Nelore (Lopes et al., 2021). This species is usually abundant in animals fed with high starch diets (O’Herrin and Kenealy, 1993) and produces formate and SCFA, such as acetate and succinate (Russell and Hespell, 1981). Formate can be reduced to CO_2_, H_2_, and CH_4_ by the action of rumen methanogens (Lovley et al., 1984), which can lead to a significant loss of feed energy and, consequently, a reduced feed efficiency.

The Christensenellaceae (gut group R-7) produces acetate and butyrate as fermentation end products in the rumen and was identified as dominant in the ileum of high-feed conversion rate (FCR) pigs (Quan et al., 2018). This taxon was also associated with methane emission in Holstein cows (Ramayo-Caldas et al., 2020) a phenotype that can negatively influence feed efficiency (Hegarty et al., 2007). Interestingly, contrary to our results with the rumen microbiome, the family Christensenellaceae was linked to Low-RFI in the stool microbiome of Angus steers (Welch et al., 2021).

Regarding the stool environment, the only bacterial ASV associated with High-RFI was classified as belonging to the Ruminococcaceae UCG-005 genus-level cluster. Although the family Ruminococcaceae is highly abundant in the stool microbiome of ruminants (Andrade et al., 2020; Dai et al., 2021), there is limited literature exploring the relationship between members of this microbiome and feed efficiency phenotypes in ruminants. Nonetheless, members of this family were correlated with the FCR trait in pigs (McCormack et al., 2017; Aliakbari et al., 2021).

### The Potential of Rikenellaceae (Gut Group RC9) as a Biomarker for Feed Efficiency

The ASVs abundancy associated with an increase of the feed efficiency trait in both rumen and stool environments were taxonomically classified as belonging to the Rikenellaceae (gut group RC9). The Rikenellaceae (gut group RC9), as other members of the Rikenellaceae family, can produce different SCFAs, such as propionate, acetate, and succinate, some with anti-inflammatory properties (Tedelind et al., 2007; Parada Venegas et al., 2019). Gut microbes associated with anti-inflammatory effects have been linked to gut health and with feed-efficient pigs, as inflammation may redirect feed energy that, otherwise, could be used for host growth and weight gain (Gardiner et al., 2020).

Besides, Welch et al. (2021) hypothesized that the link between highly feed-efficient animals and stool bacteria belonging to the Rikenellaceae family resides in the abundance of glycosaminoglycans present in their hindgut, as individuals from this family can use mucin as carbon and energy sources (Bomar et al., 2011), they could metabolize and have a competitive edge over other bacteria. Although this area is still in its infancy, recent studies have linked OTUs classified as belonging to this gut group, or to a lower taxonomic level such as the Rikenellaceae family. Species from this family have been linked to Low-RFI in the rumen of Nelore steers and in the stool microbiome of Angus steers, being also linked to Low-FCR in the stool microbiome of pigs (Quan et al., 2018; Lopes et al., 2021; Welch et al., 2021). Altogether, these results indicate a role of this specific taxon on this phenotype and suggest it as an RFI biomarker for highly efficient animals.

The GIT is a continuous and interconnected system, but the knowledge regarding the stool microbiome in ruminants is limited compared to the rumen microbiome for obvious reasons; however, it is a non-invasive sample that might represent a proxy for the rumen environment (Andrade et al., 2020). Nonetheless, identifying biomarkers for important production phenotypes in stool is advantageous as the sampling is less stressful and can be implemented in the animal routine. Understanding the biology of specific microorganisms that contribute to complex phenotypes may help to develop successful interventions for methane mitigation and feed efficiency improvement in bovines. Still, additional experiments have to be performed to assess the potential biomarkers identified in this study.

## Conclusion

The feed composition induced significant differences in both abundance and richness of ruminal and stool microbial populations in ruminants of the Nelore breed. The dietary treatment based on industrial by-products applied to one of our experimental groups influenced the microbiome diversity of bacteria and archaea but not of protozoa. ASVs were associated with RCH_4_ emission and RFI in ruminal and stool microbiomes. While ruminal ASVs are expected to influence CH_4_ emission and feed efficiency, the relationship of stool taxa with these traits might be associated with host health, through their link to anti-inflammatory compounds. Overall, the ASVs identified have the potential to be used as biomarkers for these complex phenotypes.

## Data Availability

All sequencing data are available in the NCBI Sequence Read Archive (SRA), under the bioproject number PRJNA638250.

## Supplementary Material

Supplementary Figure S1 Rarefaction curves using the Shannon index. All datasets **(A)** bacteria, **(B)** archaea, and **(C)** protozoa achieved an early saturation based on this metric. Blue indicates rarefaction curves with rumen samples, and black, with stool samples.

Supplementary Figure S2
**(A)** Comparison between observed ASV (amplicon sequence variant) metric between treatment groups showed no significant difference in protozoa populations of animals submitted to different diets. **(B)** Shannon index comparisons showed no significant difference (P<0.01) in the richness of protozoa populations. **(C)** PCoA using the rumen microbiome unweighted UniFrac distance showed no significant difference between groups (P > 0.1).

Supplementary Figure S3 Vulcan plot showing 27 differentially abundant bacterial ASVs (amplicon sequence variants) of different genera and families in the rumen with W-value >0.9.

Supplementary Figure S4 Vulcan plot showing three differentially abundant bacterial ASVs (amplicon sequence variants) of different genera and families in the stool with W-value >0.9.

Supplementary Figure S5 Vulcan plot showing eight differentially abundant archaea ASVs (amplicon sequence variants) of different species, genera, and families in the rumen with W-value >0.9.

Supplementary Figure S6 Vulcan plot showing seven differentially abundant archaea ASVs (amplicon sequence variants) of different species in the stool with W-value >0.9.

## Abbreviations

ASV, amplicon sequence variant; CLR, centered log-ratio; DA, differentially abundant; FCR, feed conversion rate; GIT, gastrointestinal tract; RCH_4_, residual methane emission; RFI, residual feed intake; and SCFA, short-chain fatty acid.
