# Supplementary figures and images for: Stool and Ruminal Microbiome Components Associated With Methane Emission and Feed Efficiency in Nelore Beef Cattle
Source: Front Genet. 2022 May 17;13:812828. doi: 10.3389/fgene.2022.812828 (PMC9152269; doi:10.3389/fgene.2022.812828)

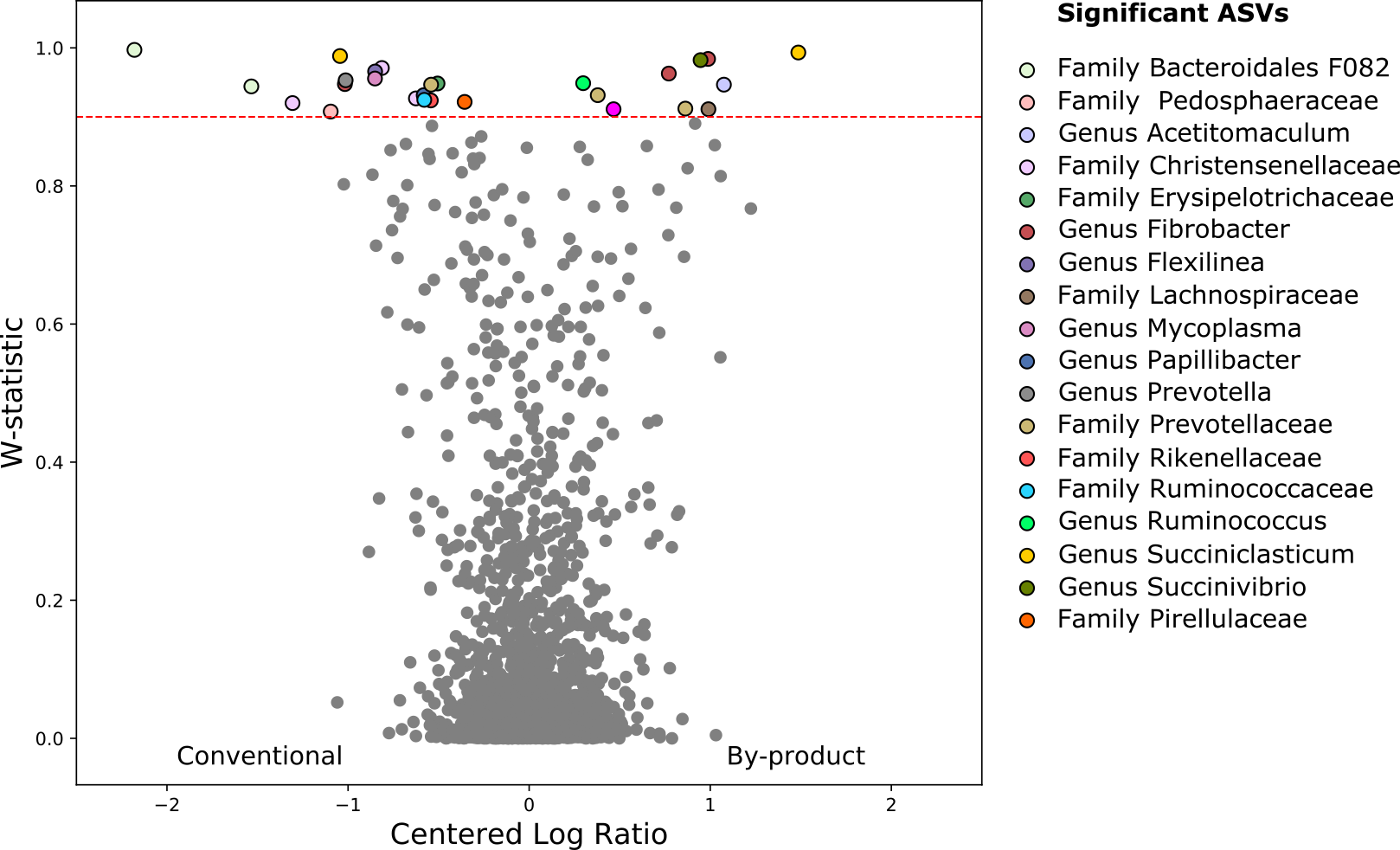

Supplement: Supplementary file 1 [file Image3.JPEG]

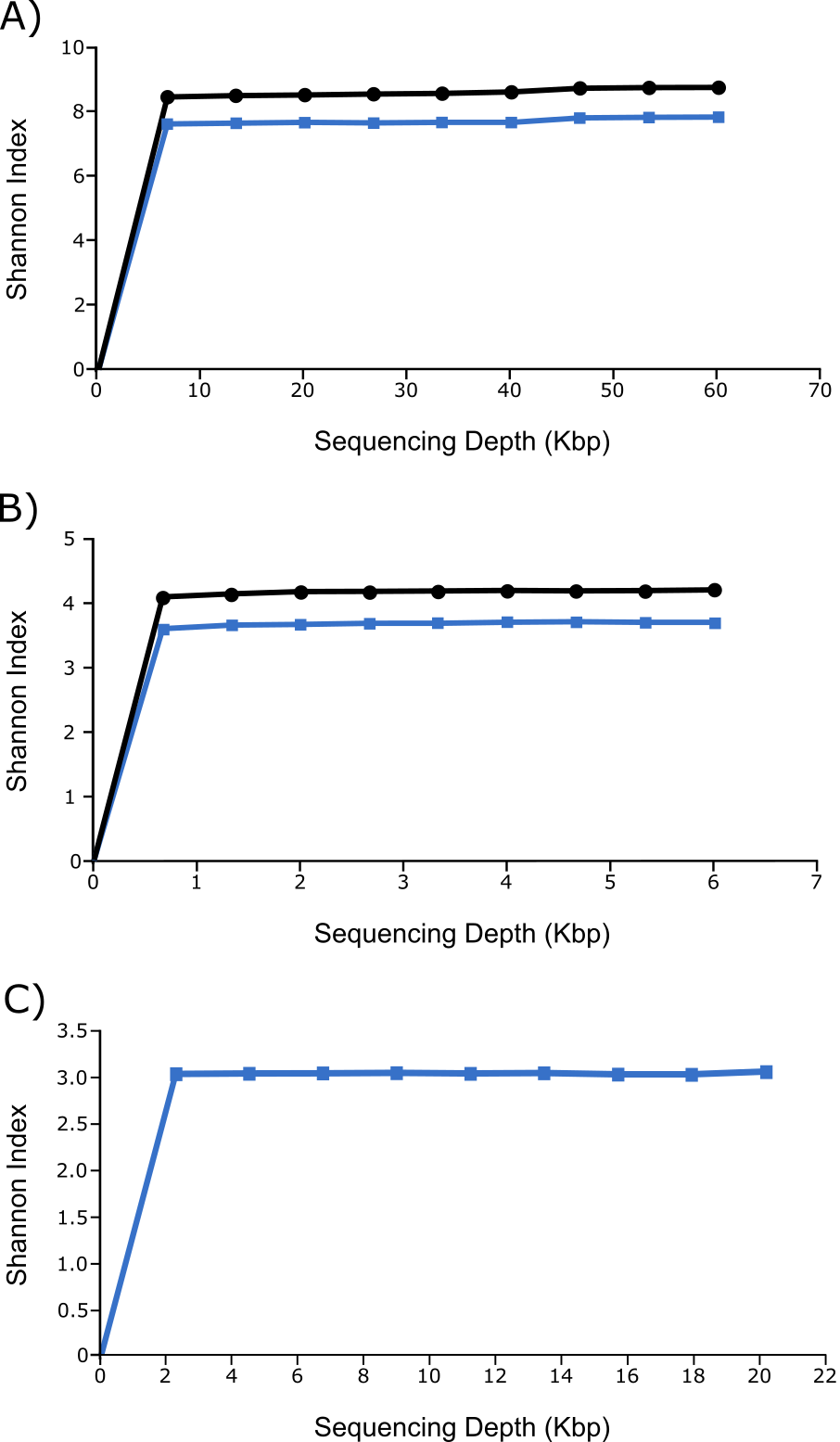

Supplement: Supplementary file 3 [file Image1.JPEG]

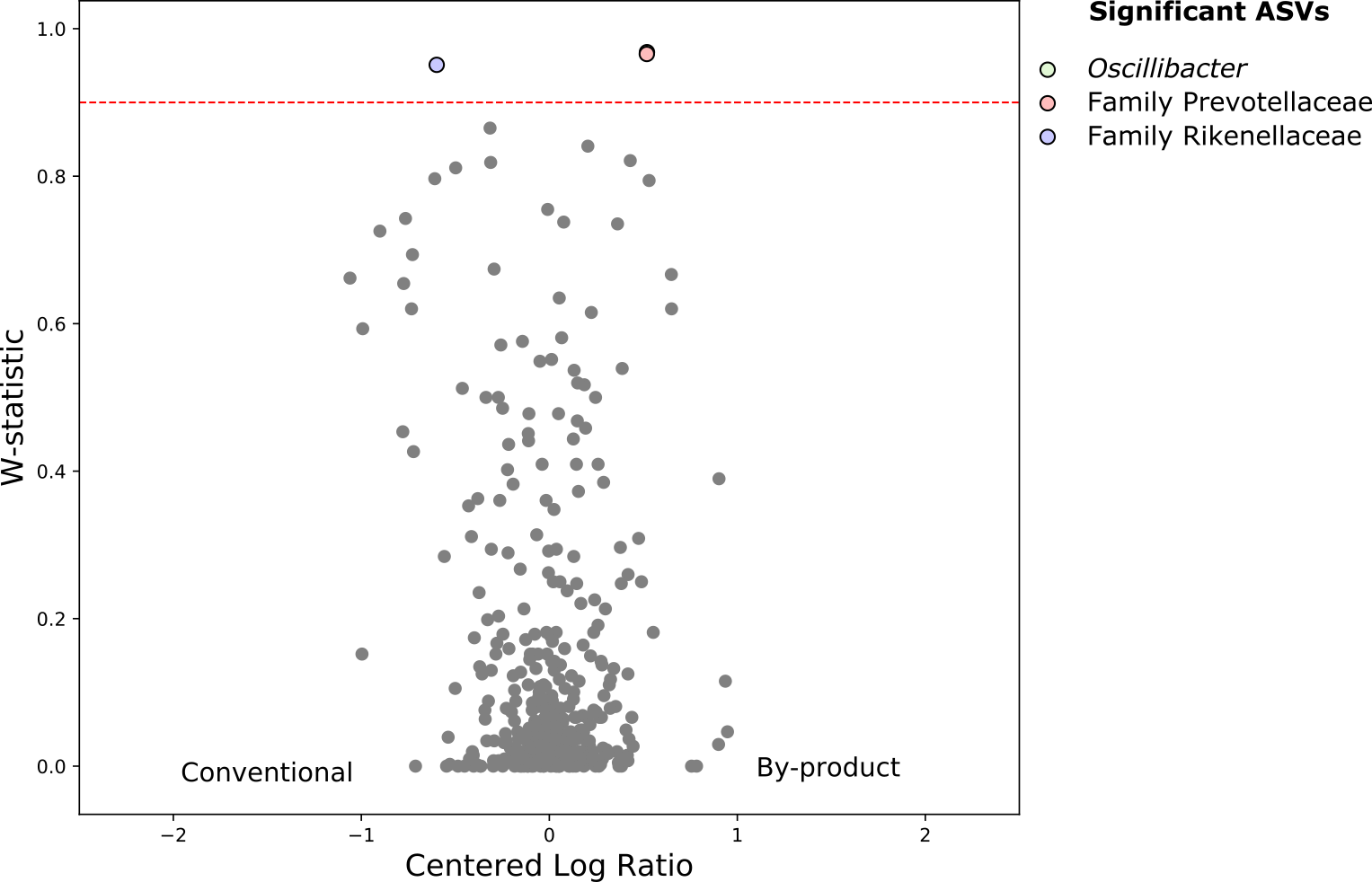

Supplement: Supplementary file 4 [file Image4.JPEG]

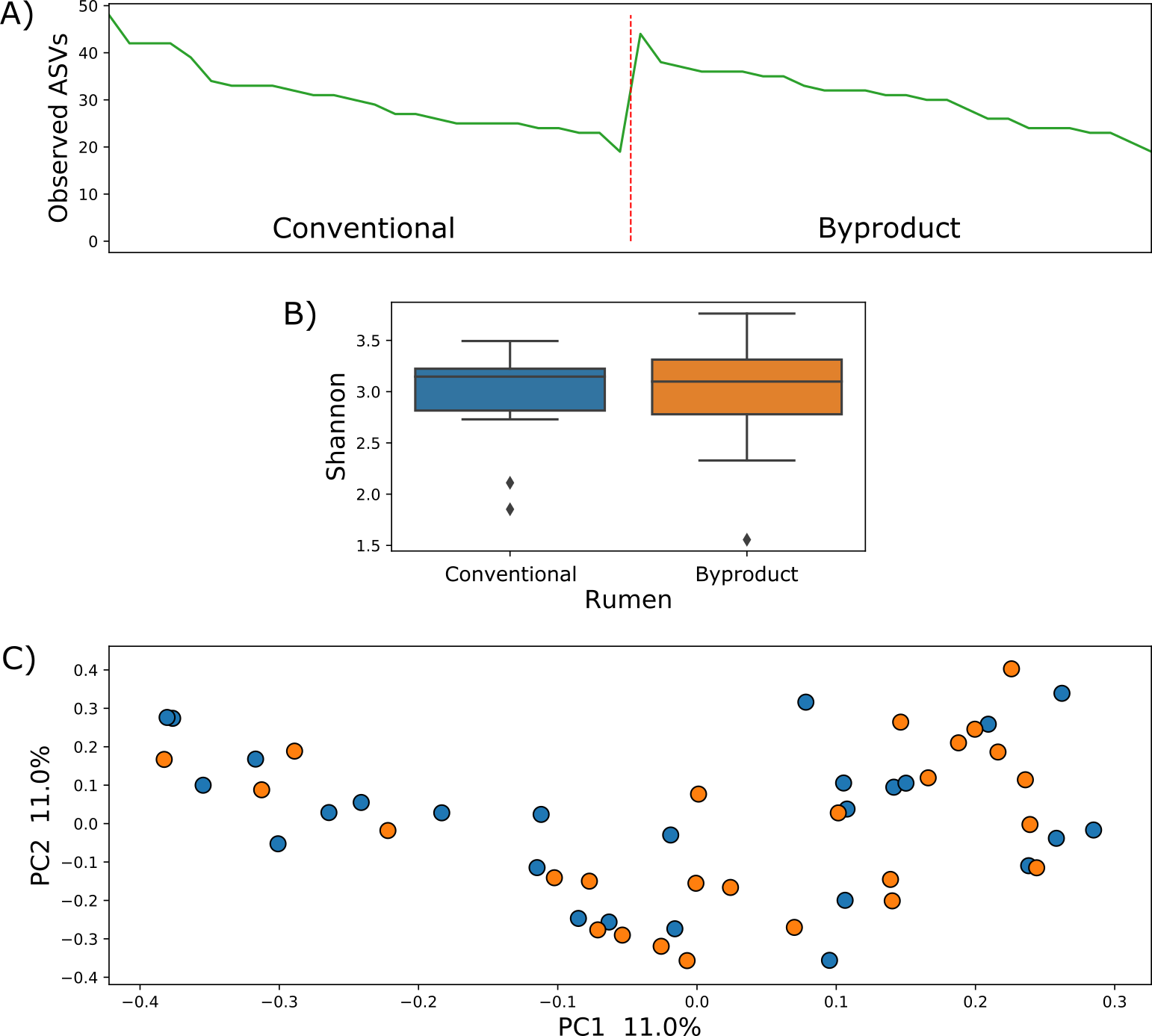

Supplement: Supplementary file 5 [file Image2.JPEG]

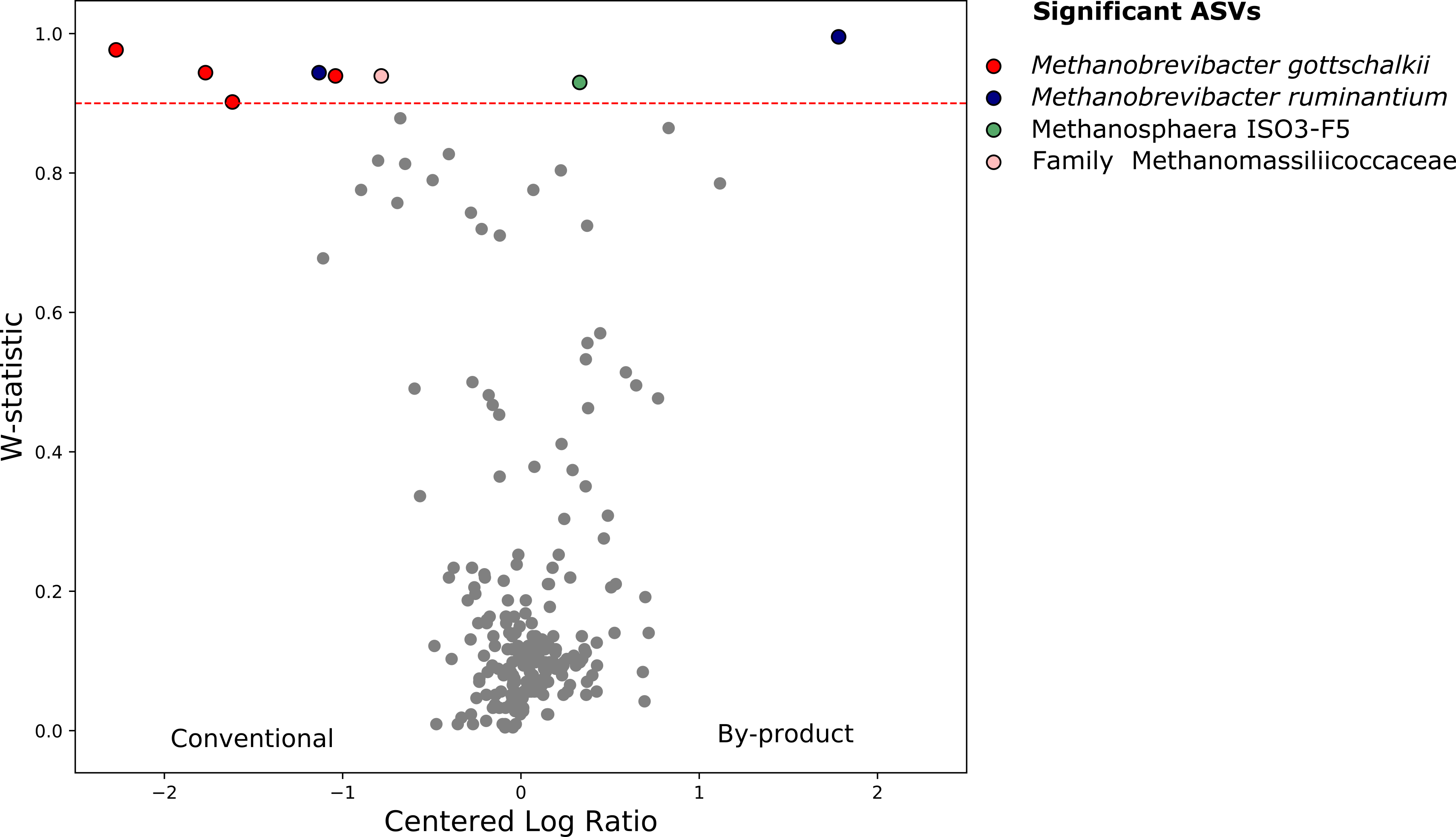

Supplement: Supplementary file 6 [file Image5.JPEG]

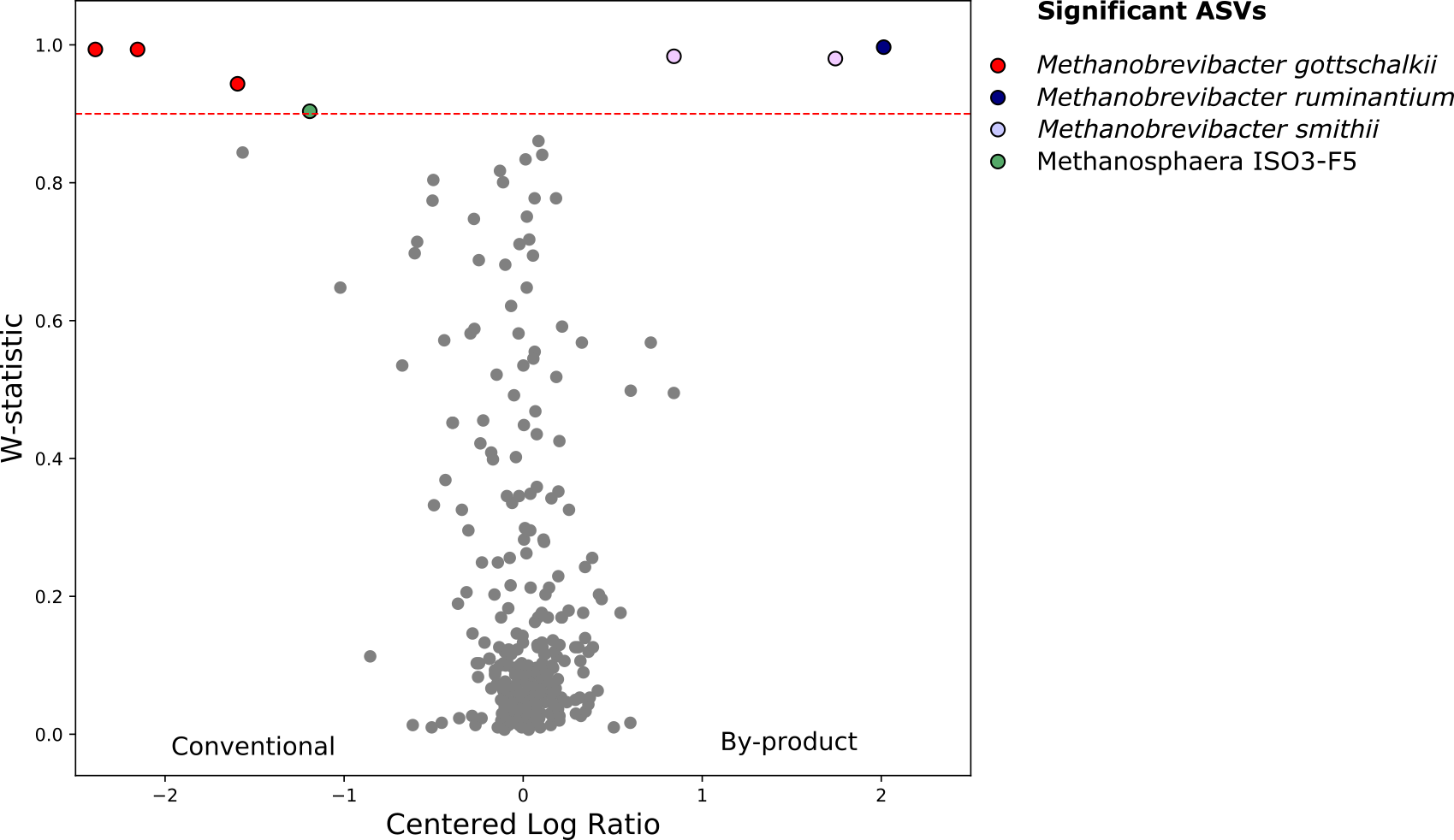

Supplement: Supplementary file 7 [file Image6.JPEG]
