# Supplementary material for: Stool and Ruminal Microbiome Components Associated With Methane Emission and Feed Efficiency in Nelore Beef Cattle
Source: Front Genet. 2022 May 17;13:812828. doi: 10.3389/fgene.2022.812828 (PMC9152269; doi:10.3389/fgene.2022.812828)
Supplement: Supplementary file 2 [file Table1.docx]

**Supplementary Table 1** – Information regarding  Animal ID,  initial weight , slaughter date, dietary treatment, Residual CH_4_ and Residual Feed intake used in this study.

| Animal ID | Initial group | Dietary | Slaughter date | RCH_4_ | RFI |
| --- | --- | --- | --- | --- | --- |
| 238 | Heavy | conventional | 16/11/2016 | 3.72 | 0.84 |
| 239 | Light | byproduct | 16/11/2016 | 21.49 | 1.39 |
| 240 | Light | byproduct | 16/11/2016 | -11.92 | -0.88 |
| 242 | Light | conventional | 17/10/2016 | 7.00 | 0.63 |
| 244 | Heavy | conventional | 17/10/2016 | -7.32 | -1.30 |
| 246 | Heavy | byproduct | 16/11/2016 | -7.23 | -1.23 |
| 464 | Light | byproduct | 16/11/2016 | -14.42 | -2.70 |
| 466 | Heavy | conventional | 17/10/2016 | -36.14 | 0.76 |
| 468 | Light | byproduct | 16/11/2016 | -14.01 | -2.20 |
| 470 | Heavy | byproduct | 17/10/2016 | 1.37 | 0.80 |
| 474 | Heavy | conventional | 17/10/2016 | 44.15 | 1.13 |
| 479 | Heavy | byproduct | 17/10/2016 | -32.67 | -0.08 |
| 482 | Heavy | conventional | 17/10/2016 | 25.17 | -2.20 |
| 483 | Light | conventional | 17/10/2016 | 34.51 | 1.56 |
| 490 | Heavy | byproduct | 17/10/2016 | 2.88 | 1.32 |
| 491 | Light | byproduct | 16/11/2016 | -5.49 | 0.09 |
| 492 | Light | conventional | 16/11/2016 | 23.85 | 0.19 |
| 494 | Light | byproduct | 17/10/2016 | 31.13 | 0.44 |
| 499 | Heavy | byproduct | 16/11/2016 | -16.94 | -2.24 |
| 500 | Heavy | conventional | 16/11/2016 | 36.79 | -0.25 |
| 502 | Light | conventional | 16/11/2016 | -34.96 | -1.85 |
| 505 | Light | conventional | 16/11/2016 | -15.40 | -1.54 |
| 506 | Light | conventional | 17/10/2016 | 7.44 | 1.18 |
| 507 | Light | byproduct | 16/11/2016 | 61.15 | 2.84 |
| 510 | Light | conventional | 16/11/2016 | 3.34 | -1.09 |
| 511 | Heavy | conventional | 16/11/2016 | 32.40 | -1.96 |
| 514 | Light | conventional | 16/11/2016 | 6.28 | -1.50 |
| 515 | Light | conventional | 17/10/2016 | 34.11 | 0.31 |
| 516 | Light | byproduct | 16/11/2016 | -19.15 | -0.76 |
| 517 | Heavy | conventional | 17/10/2016 | -12.76 | 0.80 |
| 520 | Light | byproduct | 17/10/2016 | 23.09 | -1.40 |
| 521 | Heavy | byproduct | 17/10/2016 | -22.93 | -2.78 |
| 523 | Light | conventional | 16/11/2016 | -3.22 | -0.52 |
| 1460 | Light | conventional | 16/11/2016 | 21.62 | -0.63 |
| 1462 | Heavy | byproduct | 17/10/2016 | -42.96 | 0.70 |
| 1464 | Heavy | byproduct | 17/10/2016 | 10.66 | 1.84 |
| 1468 | Heavy | conventional | 17/10/2016 | -19.81 | 2.17 |
| 1476 | Heavy | conventional | 17/10/2016 | -35.46 | 2.71 |
| 1479 | Heavy | byproduct | 17/10/2016 | 3.25 | -1.37 |
| 1480 | Light | conventional | 16/11/2016 | 15.71 | -0.02 |
| 1481 | Heavy | conventional | 16/11/2016 | -6.54 | -2.61 |
| 1485 | Heavy | byproduct | 17/10/2016 | -6.35 | 4.77 |
| 1493 | Heavy | conventional | 17/10/2016 | -28.36 | -0.14 |
| 1494 | Light | byproduct | 17/10/2016 | -18.44 | 0.39 |
| 1495 | Light | byproduct | 16/11/2016 | 27.61 | 1.06 |
| 1496 | Heavy | byproduct | 17/10/2016 | -70.63 | 0.23 |
| 1498 | Light | byproduct | 16/11/2016 | 3.30 | -2.20 |
| 1500 | Heavy | conventional | 17/10/2016 | -24.32 | -0.50 |
| 1501 | Heavy | byproduct | 16/11/2016 | 1.20 | 3.35 |
| 1502 | Light | conventional | 16/11/2016 | 48.777 | 0.34 |
| 1503 | Heavy | byproduct | 17/10/2016 | -1.90 | 0.49 |
| 1504 | Light | byproduct | 16/11/2016 | -22.66 | 1.62 |
